# Association Between Plasma Lipoprotein-Associated Phospholipase A2 and Plaque Vulnerability in TIA Patients With Unilateral Middle Cerebral Artery Stenosis

**DOI:** 10.3389/fneur.2020.574036

**Published:** 2020-10-16

**Authors:** Yiren Qin, Xiaoyan Qian, Xue Luo, Yuanfang Li, Dapeng Wang, Jianhua Jiang, Quanquan Zhang, Meirong Liu, Junhua Xiao, Yan Zhang, Shanshan Diao, Hongru Zhao

**Affiliations:** ^1^Department of Neurology, The First Affiliated Hospital of Soochow University, Suzhou, China; ^2^Department of Neurology, The First People's Hospital of Kunshan, Kunshan, China; ^3^Department of Neurology, Shiqian County People's Hospital, Tongren, China; ^4^Department of Gastroenterology, The First Affiliated Hospital of Soochow University, Suzhou, China

**Keywords:** Lp-PLA2, transient ischemic attack, middle cerebral artery, ICAs, HRMRI

## Abstract

**Background:** Plasma lipoprotein-associated phospholipase A2 (Lp-PLA2) has emerged as a novel biomarker for coronary atherosclerosis. However, the association between Lp-PLA2 and plaque vulnerability in atherosclerosis of cervicocerebral arteries remains poorly defined, especially for intracranial atherosclerotic stenosis (ICAS). We aimed to investigate the association between Lp-PLA2 and plaque vulnerability in transient ischemic attack (TIA) patients with unilateral middle cerebral artery stenoses (MCAs).

**Methods:** In this study, a total of 207 patients were enrolled from April 2017 to April 2020. Clinical data were collected, and MCA plaques were examined with high-resolution magnetic resonance imaging (HRMRI). Baseline characteristics of patients were collected during hospitalization. Statistical comparisons were performed using Pearson's chi-squared test, Mann–Whitney U test, and the Breslow–Day/Tarone's test for the determination of heterogeneity in different age strata. Multivariate binary logistic analysis was used to investigate the potential independent predictors that were highly correlated to plaque vulnerability.

**Results:** The results showed that a high Lp-PLA2 level (>221 ng/ml) was associated with plaque vulnerability in TIA patients with unilateral MCAs. High Lp-PLA2 was independently associated with plaque vulnerability in patients ≤ 60 years old [multivariate adjusted odds ratio (OR) = 9.854; 95% CI, 2.458–39.501] but not in patients >60 years old (multivariate adjusted OR = 1.901; 95% CI, 0.640–5.650). Predictors of plaque vulnerability in different age strata were also different.

**Conclusion:** Lp-PLA2 levels may be correlated to plaque vulnerability in TIA patients with unilateral MCAs and might be a diagnostic biomarker for plaque vulnerability in this kind of patients, especially for ones aged ≤ 60 years old.

## Introduction

Atherosclerosis (AS) is a kind of chronic inflammatory disease developed by heterogeneous causes (1). It plays an important role in the pathogenesis of the ischemic cerebrovascular disease. Plasma lipoprotein-associated phospholipase A2 (Lp-PLA2) has emerged as a novel inflammatory biomarker, which is involved in the initiation and progression of AS (2). Therefore, it is widely used as an inflammation predictor of atherosclerotic plaque in recent years (3). Previous studies have suggested that high expression of Lp-PLA2 correlated with poorer clinical outcome (4), increased cerebral infarction (5), and severer stenosis (6). However, the association between Lp-PLA2 and plaque vulnerability in atherosclerosis of cervicocerebral arteries, especially in ICAS patients has not been widely explored. For the last few years, high-resolution magnetic resonance imaging (HRMRI) was applied as a crucial noninvasive technique for the clinical determination of ICAS. It can objectively exhibit intracranial arterial wall morphology and atherosclerosis plaque characteristics by obtaining morphological measurements for atherosclerosis plaque composition (7, 8). However, it is much more expensive compared with traditional techniques, and is difficult to be applied in primary hospitals (9). Therefore, we aimed to investigate the association between Lp-PLA2 and plaque vulnerability in transient ischemic attack (TIA) patients with middle cerebral artery stenoses (MCAs) and explored whether Lp-PLA2 could be a potential diagnostic biomarker for plaque vulnerability in intracranial atherosclerotic artery.

## Materials and Methods

### Study Population

This double-center study was reviewed and approved by the Ethics Committees of The First Affiliated Hospital of Soochow University and the Ethics Committees of The First People's Hospital of Kunshan, respectively. It was performed as a retrospective analysis of prospectively collected data between April 2017 and April 2020. Two hundred seven TIA patients with unilateral MCAs were recruited into our study. The inclusion criteria were as follows: (1) aged from 18 to 90 years; (2) diagnosed of first-ever TIA; (3) diagnosis of TIA fulfilled the World Health Organization (WHO) criteria with a complete clinical remission within 24 h, and the time-based diagnosis has been verified in diffusion-weighted MRI demonstrating no relevant ischemic lesions; and (4) with CT angiography (CTA) evidence of unilateral MCAs of 70% or more and referred for HRMRI. Exclusion criteria were as follows: (1) previous antiplatelet, anticoagulant, thrombolytic, and statins therapies; (2) previous cerebral infarction; (3) previous cranial bleeding or hemorrhage; (4) previously diagnosed with thrombocytopenia or tumor, severe renal, hepatic, heart dysfunction; (5) previous heart disease (e.g., atrial fibrillation, coronary artery disease); (6) internal carotid artery dissection or cerebral vasculitis; (7) occlusion of unilateral MCA or acute cerebral infarction; (8) bilateral MCA stenosis; (9) severe stenosis or vulnerable plaque in the carotid artery; and (10) recurrent TIA.

### Data Collection

All patients received the National Institutes of Health Stroke Scale (NIHSS) assessment by a qualified neurologist according to the symptom. Patients were etiologically assessed with other hospital workups including transcranial cerebral Doppler (TCD), echocardiography, and Holter monitor. Baseline characteristics of patients that may be associated with stroke risk were collected during hospitalization. MCA-M1 plaques were examined with HRMRI to assess the vulnerability of the plaques. Seven lipid parameters included total cholesterol (TC), total triglycerides (TG), high-density lipoproteins (HDLs), low-density lipoproteins (LDLs), apolipoprotein A (APOA), apolipoprotein B (APOB), and lipoprotein a (LPa).

### Measurement of Lp-PLA2

Whole venous blood was drawn in tubes containing ethylene diamine tetraacetic acid (EDTA). The blood sample was centrifuged and was separated and placed in microtubes. Samples for measurement of Lp-PLA2 mass were handled simultaneously by a professional technician using the human Lp-PLA2 enzyme ELISA kit (Hotgen, Beijing, China). All protocols, e.g., precoat of specific antibody, incubation with primary and biotin-conjugated antibodies to Lp-PLA2, and optical density measurements, were performed according to the manufacturer's instructions.

### High-Resolution MRI Protocol

HRMRI was performed using a 3.0-T MRI scanner (Ingenia, Philips Healthcare, Best, the Netherlands) with dedicated eight-channel head and neck unite coil. A standardized MR protocol was performed including routine MRI images and three-dimensional (3D) time-of-flight (TOF) magnetic resonance angiography (MRA). The MCA-M1 segment for HRMRI scanning was obtained by a neuroradiologist. The sequence parameters were as follows: First, pre- and post-contrast T1WI image scanning used a turbo spin echo (TSE) measurement of the black blood two-dimensional (2D); repetition time (TR)/echo time (TE), 500/8.8 ms; TSE factor, 12; band width (BW), 393.1 MHz; and number of average (NEX), 1. Next, the high-resolution T2WI image scanning was acquired also using TSE sequence; parameters were as follows: TR/TE, 2,500/271 ms; TSE factor, 100; BW, 561.9 MHz; and NEX, 2. After HR-T2WI, the high-resolution PDWI image scanning was acquired also using TSE sequence; parameters were as follows: TR/TE, 2,000/40 ms; TSE factor, 60; BW, 425.2 MHz; and NEX, 1. Both acquisitions were run with a field of view (FOV) of 13 cm, matrix size of 256, slice thickness of 0.5 mm, and non-gap, pixel size was 0.5 × 0.5 × 0.5. Scan direction was parallel or perpendicular to the M1 segment. Three minutes later, contrast agent (gadopentetate dimeglumine, Gd-DTPA, Magnevist; Bayer Schering Pharma, Berlin, Germany) was injected intravenously with a dose of 0.1 mmol/kg and flowrate of 2 ml/s.

### Image Analysis

**Figure 1:**
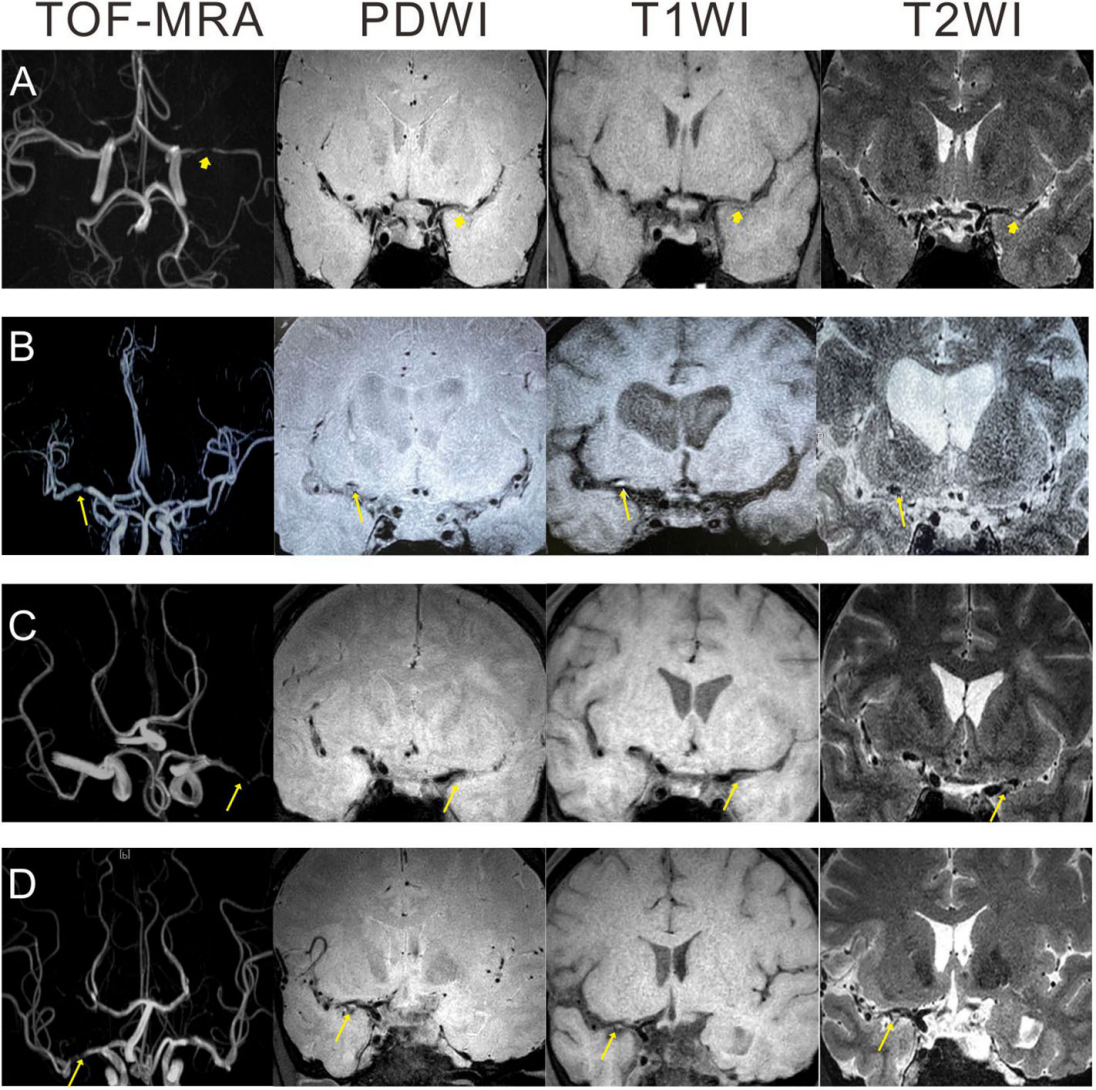
Illustration of HRMRI images showing atherosclerotic plaques in patients with unilateral MCAs. **(A)** A 30 year-old man with non-vulnerable plaque: no high signal intensity on PDWI, T1WI, and T2WI. **(B)** A 70 year-old man with intraplaque hemorrhage: isointensity on PDWI, eccentric high signal intensity on T1WI, and hypointensity on T2WI. **(C)** A 42 year-old man with plaque enhancement: isointensity on PDWI, eccentric slight hyperintensity on T1WI, and slight hyperintensity on T2WI. **(D)** A 47 year-old man with heterogeneous signal intensity: mixed signal intensity on PDWI, T1WI, and T2WI. Arrowhead: non-vulnerable plaque; arrow: vulnerable plaque. HRMRI, high-resolution MRI; PDWI, proton density weighted imaging; TOF, time-of-flight; T2WI, T2-weighted imaging; T1WI, T1-weighted imaging; MCAs, middle cerebral artery stenosis.

All images of the MCA plaques were reviewed and analyzed on a workstation by two well-trained neuroradiologists (one >10 years' experience and one >5 years' experience) independently blinded to the patient's clinical details. The differences between two neuroradiologists were solved by a senior consensus. The distributions, numbers, and locations of plaques were evaluated. Lipid-rich core, hemorrhage in the plaque, fibrous cap rupture, and/or calcified nodules protruding into the lumen were evaluated as previously described (10, 11). In summary, based on the previous researches (12–14), plaque vulnerability was defined following the three major criteria: (1) intraplaque hemorrhage (IPH, >150% intensity compared with the adjacent normal gray matter), (2) plaque enhancement, and (3) plaques with heterogeneous signal intensity. The illustrative HRMRI images were demonstrated in Figure 1.

### Statistical Analysis

Statistical analysis was performed with the SPSS version 24.0 program. The sample size determination of our study was done by PASS 15.0 software. Direct studies for patients with unilateral MCAs were not available to support a comparison between the normal and high levels of Lp-PLA2. Based on the preliminary data of our study, we estimated the percentage of patients with plaque vulnerability at normal and high levels of Lp-PLA2 at ~20 and 40%, respectively. Meanwhile, we assumed that the group allocation of the normal and high levels of Lp-PLA2 was 1. Thus, a sample size of 180 patients was calculated as the minimum number required to yield a power of 80% with a two-sided significance level of 5%. Data are presented as median (IQR) for continuous variables and counts (percentages) for categorical data. Comparisons of patient characteristics were performed with the Mann–Whitney U test or Pearson's χ^2^ test. Multivariate logical regression analysis was used to detect the potential predictors of plaque vulnerability. The homogeneity assessment among different layers of age was determined by Breslow–Day and Tarone's test. The definition of low/high LP-PLA2 and cutoff of LP-PLA2 were concluded by receiver operating characteristic (ROC) curve and area under curve (AUC). A value of *p* < 0.05 (two-sided) was considered to be statistically significant.

## Results

### Demographic Comparisons of the Studied Patients With Vulnerable and Non-vulnerable Plaque

We enrolled 207 eligible TIA patients with unilateral MCAs who received HRMRI. Baseline characteristics of patients diagnosed with a vulnerable and non-vulnerable plaque are summarized in Table 1. The levels of HDL, APOB, and Lp-PLA2 were statistically different between patients with vulnerable and non-vulnerable plaque (Mann–Whitney test, *p*-values were 0.005, 0.030, <0.001, respectively). The percentage of smoke in patients with a vulnerable plaque was statistically higher than that with non-vulnerable plaque (57.1 vs. 39.4%, *p* = 0.015). The illustrative HRMRI images for patients diagnosed with a vulnerable and non-vulnerable plaque are provided in Figure 1.

**Table 1:** Characteristics of the studied patients with vulnerable and non-vulnerable plaque.

**Characteristics**	**VP Non-VP**	***r-***	**Z or χ^2^**	***p-***
	**Median (IQR) or n (%)**	**value**		**value**
Age	61 (19) 59 (16)	−	−0.345	0.730
Admission glucose (mmol/L)	5.61 (2.21) 5.69 (1.79)	−	−1.077	0.282
Admission SBP (mmHg)	151 (38.75) 151 (31.5)	−	−0.932	0.351
Admission DBP (mmHg)	84.5 (25) 85 (21.5)	−	−1.070	0.285
Admission PP (mmHg)	64 (32) 64 (29)	−	−0.222	0.824
HDL (mmol/L)	1.17 (0.38) 1.27 (0.34)	−	−2.801	0.005
LDL (mmol/L)	2.63 (1.37) 2.69 (1.24)	−	−0.384	0.701
TC (mmol/L)	4.27 (1.46) 4.45 (1.70)	−	−1.208	0.200
TG (mmol/L)	1.27 (0.78) 1.22 (0.87)	−	−0.877	0.381
APOA (g/L)	1.15 (0.33) 1.18 (0.28)	−	−1.065	0.287
APOB (g/L)	0.89 (0.42) 0.82 (0.33)	−	−2.170	0.030
LPa (mg/L)	156 (206.25) 135 (155)	−	−1.004	0.315
NIHSS	5 (6) 5 (6)	−	−0.527	0.598
Lp-PLA2 (ng/ml)	275 (144.5) 206 (72)	−	−5.774	<0.001
HBP	55 (78.6) 111 (81.0)	−0.029	0.175	0.676
DM	23 (32.9) 41 (29.9)	0.030	0.186	0.666
Smoke	40 (57.1) 54 (39.4)	0.168	5.873	0.015
Drink	16 (22.9) 29 (21.2)	0.019	0.078	0.780
Sex, male	44 (62.9) 76 (55.5)	0.071	1.036	0.309

*VP, vulnerable plaque; TC, total cholesterol; TG, total triglycerides; HDL, high-density lipoproteins; LDL, low-density lipoproteins; APOA, apolipoprotein A; APOB, apolipoprotein B; LPa, lipoprotein a; NIHSS, the National Institutes of Health Stroke Scale; HBP, high blood pressure; DM, diabetes mellitus*.

### Association of Lp-PLA2 With Plaque Vulnerability

**Figure 2:**
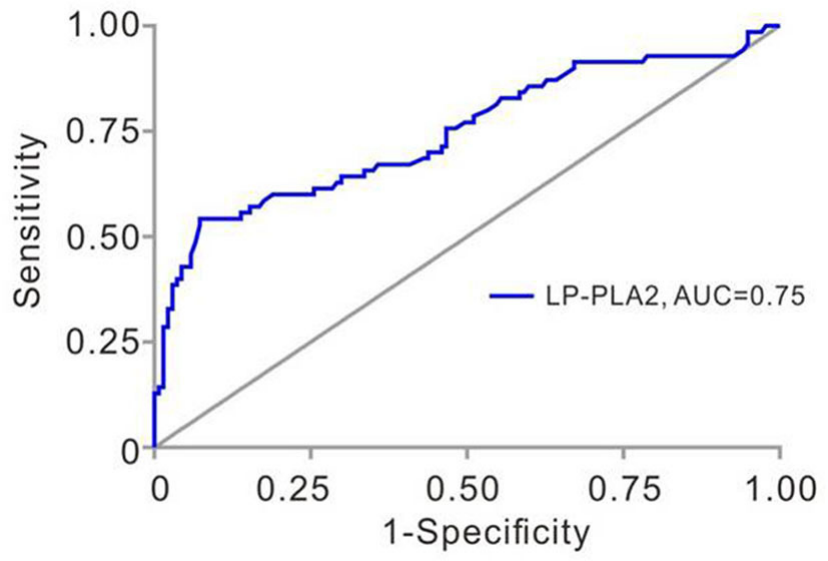
Receiver operating characteristic (ROC) analysis of Lp-PLA2 in TIA patients with unilateral MCAs. Area under the ROC curve for Lp-PLA2 was 0.746 (95% CI, 0.669–0.822, *p* < 0.001). Lp-PLA2, lipoprotein-associated phospholipase A2; TIA, transient ischemic attack; MCAs, middle cerebral artery stenoses.

We next determined the sensitivity and specificity of Lp-PLA2 levels for the diagnosis of plaque vulnerability by using ROC analysis. The AUC of Lp-PLA2 levels for plaque vulnerability was 0.7455 (95% CI, 0.6690–0.8219; *p* < 0.001). The Lp-PLA2 level of 221 ng/ml was determined as the optimal cutoff value to predict plaque vulnerability. The sensitivity and specificity were 54.3 and 92.7%, respectively (Figure 2). Thus, we classified Lp-PLA2 into a categorical variable (high Lp-PLA2 > 221 ng/ml) and analyzed the association of high Lp-PLA2 with plaque vulnerability in different logistic models. In crude analysis, high Lp-PLA2 was associated with plaque vulnerability statistically (OR = 2.956; 95% CI, 1.616–5.407). After age–sex, seven items of lipidemia, multivariate adjustments, and high Lp-PLA2 were still statistically related to the plaque vulnerability (Table 2).

**Table 2:** Odds ratios for diagnosis of plaque vulnerability in different models.

	**High Lp-PLA2**	***p-*value**
	**OR (95% CI)**	
Crude	2.956 (1.616–5.407)	<0.001
Age and sex	2.859 (1.551–5.269)	<0.001
Lipidemia (seven)	2.902 (1.504–5.601)	0.001
Multivariate	2.718 (1.347–5.486)	0.005

### Effect of Lp-PLA2 on Plaque Vulnerability Among Different Age Strata

To identify the optimal cutoff of age for high Lp-PLA2 to predict plaque vulnerability, age was stratified into a dichotomous variable, and homogeneity test for the effect of high Lp-PLA2 among different age strata was analyzed every 10 years. As shown in Table 3, the Breslow–Day/Tarone's test revealed a most statistically homogeneity in age 60 (χ^2^ = 6.083 and *p* = 0.014, and χ^2^ = 6.070 and *p* = 0.014, respectively). We then performed a binary logistic analysis to investigate the potential interaction between age and high Lp-PLA2. The interaction of age and high Lp-PLA2 was statistically significant (OR = 4.720; 95% CI, 1.358–16.400, *p* = 0.015). This indicated that the effect of high Lp-PLA2 on plaque vulnerability might be diminished with age.

**Table 3:** Homogeneity determination of lipoprotein-associated phospholipase A2 (Lp-PLA2) across the age strata and interaction of Lp-PLA2 with age.

		**Chi-squared**			***p*****-value**		
**Tests of homogeneity of the odds ratio**							
Breslow–Day		6.083			0.014		
Tarone's		6.070			0.014		
**Variables**		**High Lp-PLA2**	**Normal Lp-PLA2**	**χ2**	***r-*****value**	***p-*****value**	**OR (95% CI)**
**Interaction test of Lp-PLA2 with age**							
≤ 60 (*n* = 114)	VP (*n* = 34)	26 (49.1)	8 (13.1)	17.504	0.392	<0.001	6.380 (2.548–15.976)
> 60 (*n* = 93)	VP (*n* = 36)	21 (42.0)	15 (34.9)	0.493	0.073	0.482	1.352 (0.582–3.137)
Test for interaction		–	–	–	–	0.015	4.720 (1.358–16.400)

*VP, vulnerable plaque*.

### Predictors of Plaque Vulnerability Analyzed by Multivariate Binary Regression Model in Patients Aged ≤ 60 or >60 Years

We then performed binary logistic regression analyses in patients aged ≤ 60 or >60 years. As summarized in Tables 4, 5, we found that high Lp-PLA2 was an important independent predictor for plaque vulnerability in patients ≤ 60 years (Table 4, B = 2.288, OR = 9.854, 95% CI, 2.458–39.501, *p* = 0.001). However, in patients >60 years, this predictability achieved no statistical significance (Table 5; B = 0.642; OR = 1.901; 95% CI, 0.640–5.650, *p* = 0.248). In addition, HDL, APOB, and smoke were independent predictors of plaque vulnerability in patients ≤ 60 years (Table 4; OR = 0.020, 1.618, 4.793; 95% CI, 0.001–0.441, 1.113–2.352, 1.446–15.891, respectively), and in patients >60 years, NIHSS and smoke were important predictors (Table 5; OR = 2.287, 2.719; 95% CI, 1.218–4.293, 1.196–6.179, respectively).

**Table 4:** Multivariate logistic regression analysis of variables associated with plaque vulnerability in studied patients aged ≤ 60 years old.

**Variables**	**B**	**S.E**.	**Wald**	***p*-value**	**OR (95% CI)**
High Lp-PLA2	2.288	0.708	10.431	0.001	9.854 (2.458–39.501)
TC	−1.149	0.987	1.356	0.244	0.317 (0.046–2.193)
TG	−0.446	0.344	1.679	0.195	0.640 (0.326–1.257)
HDL	−3.912	1.579	6.141	0.013	0.020 (0.001–0.441)
LDL	1.050	1.229	0.730	0.393	2.858 (0.257–31.791)
APOB	0.481	0.191	6.363	0.012	1.618 (1.113–2.352)
APOA	−0.242	0.365	0.438	0.508	0.785 (0.384–1.606)
LPa	−0.020	0.024	0.649	0.420	0.981 (0.935–1.029)
NIHSS	−0.056	0.087	0.418	0.518	0.945 (0.797–1.121)
HBP	1.323	0.883	2.247	0.134	3.755 (0.666–21.177)
DM	0.586	0.717	0.668	0.414	1.797 (0.441–7.321)
Smoke	1.567	0.612	6.567	0.010	4.793 (1.446–15.891)
Sex, male	0.916	0.747	1.505	0.220	2.499 (0.578–10.794)

*TC, total cholesterol; TG, total triglycerides; HDL, high density lipoproteins; LDL, low density lipoproteins; APOA, apolipoprotein A; APOB, apolipoprotein B; LPa, lipoprotein a; NIHSS, the National Institutes of Health Stroke Scale; HBP, high blood pressure; DM, diabetes mellitus*.

**Table 5:** Multivariate logistic regression analysis of variables associated with plaque vulnerability in studied patients aged > 60 years old.

**Variables**	**B**	**S.E**.	**Wald**	***p*-value**	**OR (95% CI)**
High Lp-PLA2	0.642	0.556	1.336	0.248	1.901 (0.640–5.650)
TC	−0.029	0.097	0.091	0.763	0.971 (0.803–1.174)
TG	−0.053	0.081	0.426	0.514	0.949 (0.809–1.112)
HDL	−1.207	0.959	1.584	0.208	0.299 (0.046–1.960)
LDL	−0.651	1.163	0.314	0.575	0.521 (0.053–5.093)
APOB	0.353	0.214	2.730	0.098	1.424 (0.936–2.164)
APOA	0.183	0.542	0.114	0.736	1.201 (0.415–3.478)
LPa	0.016	0.015	1.089	0.297	1.016 (0.986–1.047)
NIHSS	0.827	0.321	6.623	0.010	2.287 (1.218–4.293)
HBP	0.190	0.701	0.073	0.787	1.209 (0.306–4.772)
DM	−0.193	0.674	0.082	0.775	0.825 (0.220–3.091)
Smoke	1.000	0.419	5.703	0.017	2.719 (1.196–6.179)
Sex, male	0.447	0.558	0.642	0.423	1.564 (0.524–4.665)

*TC, total cholesterol; TG, total triglycerides; HDL, high density lipoproteins; LDL, low density lipoproteins; APOA, apolipoprotein A; APOB, apolipoprotein B; LPa, lipoprotein a; NIHSS, the National Institutes of Health Stroke Scale. HBP, high blood pressure; DM, diabetes mellitus*.

### ROC Analyses for Plaque Vulnerability in Patients Aged ≤ 60 or >60 Years

**Figure 3:**
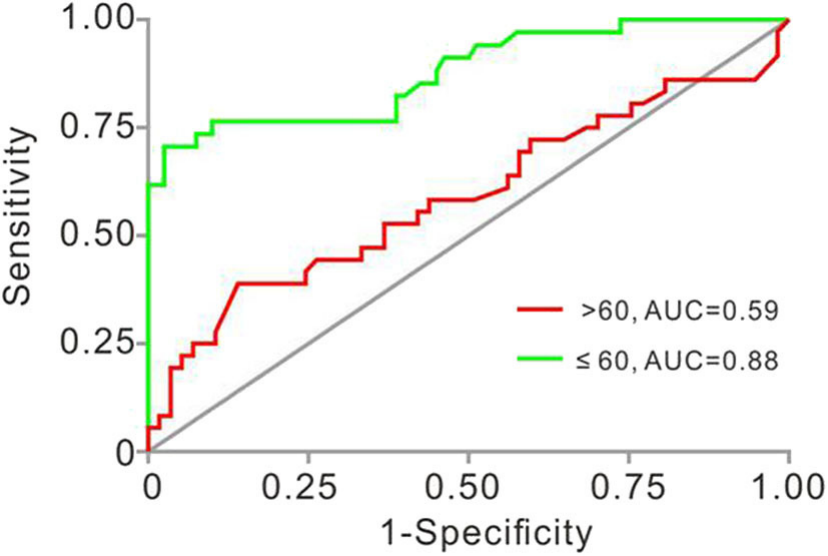
Receiver operating characteristic (ROC) analyses of Lp-PLA2 in studied patients aged ≤ 60 and >60 years old. AUC for Lp-PLA2 was 0.878 (≤ 60, 95% CI, 0.802–0.954, *p* < 0.001) and 0.5899 (>60, 95% CI 0.4645–0.7153, *p* = 0.1457). AUC, area under the ROC curve; Lp-PLA2, lipoprotein-associated phospholipase A2.

At last, we investigated the sensitivity and specificity of Lp-PLA2 levels for the diagnosis of plaque vulnerability in different age strata. In patients ≤ 60 years, the AUC of Lp-PLA2 levels for plaque vulnerability was 0.8779 (95% CI, 0.8023–0.9536, *p* < 0.001). The Lp-PLA2 level of 221 ng/ml was determined as the optimal cutoff value to predict plaque vulnerability. The sensitivity and specificity were 70.6 and 97.5%, respectively. However, there was no statistical predictive value of Lp-PLA2 [AUC, 0.5899 (95% CI, 0.4645–0.7153), *p* = 0.1457] in patients >60 years old with ROC analysis (Figure 3).

## Discussion

Intracranial atherosclerotic stenosis is one of the most common causes of cerebral infarction (CI) and TIA (15). Studies have shown that ICAS is predominant in Asian, African, and Hispanic origin, whereas extracranial arterial stenosis (ECAS) is more prevalent in Caucasians (16). In China, one-third case of ischemic stroke was related to ICAS, in which large intracranial arteries, e.g., MCA were involved (17). Previous studies have shown that about 10% of cases of TIA and 30–50% cases of CI were caused by MCA stenosis (18), and malignant MCA infarction is correlated with up to 80% mortality in the first week (19). Therefore, it is very important to evaluate MCA as accurately as possible (20). Conventionally, TCD, MRA, CTA, and digital substraction angiography (DSA) are performed to evaluate intracranial arterial stenosis. However, they cannot exhibit the wall morphology and atherosclerosis objectively. Although intracranial arterial stenosis is related to the occurrence of ischemic events (21), current studies have shown that the occurrence of ischemic events in patients with symptomatic intracranial atherosclerosis is more relevant to the characteristics of a plaque rather than arterial stenosis by working with HRMRI (22). Therefore, it may be more important to evaluate plaque vulnerability in terms of predicting the risk of ischemic events rather than simply assess arterial stenosis (23).

Recently, HRMRI has been used as a promising modality to depict the intracranial artery wall and interpret plaque characteristics by combining a multitude of sequences. In the past few years, it has been hypothesized that the pathophysiology of intracranial atherosclerosis parallels that in the carotid artery by using HRMRI (14, 20, 24). Previous histological comparison also pointed that the characteristics of plaque within the intracranial arteries were the same as those in carotid arteries, such as intraplaque hemorrhage, fibrous cap, and large lipid-rich necrotic core (20, 25). However, several current studies have shown slight discrepancies of intracranial plaque from those in carotid arteries (26). For example, fibrous cap is much thinner and is demonstrated with more ambiguous signal intensity than those in carotid plaques (27). Therefore, intracranial fibrous cap rupture may not be sensitively identified by HRMRI (28). With regard to intraplaque hemorrhage, the lower prevalence in MCA indicated that it is still far away from the “true” promising tool due to the smaller hemorrhagic volume and very complicated and muddled hemorrhagic components (29). Furthermore, some other limitations also influence its application in the evaluation of intracranial arterial plaques. HRMRI highly depends on its long acquisition time, coil quality, and resolution. Meanwhile, it is limited by its cost and availability, especially in primary hospitals (9). Therefore, it is of great significance to find a convenient, quick-applied, and relatively accurate technique to evaluate the plaque vulnerability of intracranial arteries as a supplementary diagnostic method.

AS is a chronic inflammatory disease. There are many inflammatory cytokines involved in the entire process of atheroma formation and development (30). Lp-PLA2 has emerged as a novel biomarker of inflammation. Approximately 80% of Lp-PLA2 circulates bound to LDL, whereas the other 20% is bound to HDL and very low-density lipoprotein (VLDL) (31). Lp-PLA2 can hydrolyze oxPL at the surface of LDL, which produces a variety of cytokines, adhesion factors, and promotes the formation of atherosclerotic plaques (32). Meanwhile, histopathological examinations have shown that Lp-PLA2 is highly expressed in the late necrotic core of atheromatous plaque, suggesting that it is involved in the formation and development of vulnerable atherosclerotic plaques (33). Theoretically, the determination of plasma Lp-PLA2 can predict the vulnerability of plaque. Nevertheless, previous studies were mostly focused on the correlation between carotid plaque vulnerability and Lp-PLA2 (6, 34). It is well-known that plaque vulnerability of carotid, especially in extracranial carotid, can be assessed by carotid ultrasound. In contrast, due to the presence of the skull, the intracranial artery can only be evaluated for the blood flow velocity with TCD and cannot be evaluated for plaque vulnerability directly (35). Thus, it is more valuable to investigate the correlation between Lp-PLA2 and plaque vulnerability in intracranial atherosclerotic arteries. In this study, our results suggested that the Lp-PLA2 level in patients with intracranial vulnerable plaque is statistically higher than that with non-vulnerable plaque, which is in accordance with the previous studies (36, 37). We also found that a high Lp-PLA2 level (>221 ng/ml) was associated with MCA plaque vulnerability in patients ≤ 60 years but not in patients >60 years old. This may be because atherosclerosis is more prevalent in the elderly than in young adults. On the other hand, it may also be related to the decreased sensitivity of elderly patients to atherosclerotic inflammation (38).

Finally, some important limitations merit consideration: First, the sample size of studied patients was relatively small. Larger sample, prospective, multicenter studies or randomized trials of potent reversible pharmacological Lp-PLA2 inhibitors are further needed to clarify this issue in the future. Second, we only used qualitative approaches to discriminate plaque types. The visual evaluation was not absolutely objective, although the assessment was blinded to clinical information with senior consensus. Currently, quantitative measurements of signal intensity in MCAs offer standardization to distinguish the differences between vulnerable and non-vulnerable plaque by HRMRI (39). Until recently, MR signal features can be identified for the stenosis, plaque burden, and vulnerability (stenotic lumen area, stenotic wall area, plaque eccentricity, and plaque length), minimum luminal area, intraplaque hemorrhage, enhancement ratio, and dispersion of signal intensity (coefficient of variation) in a quantitative manner (40). Thus, this issue merits further quantitative validation. Third, the level of plasma Lp-PLA2 was assessed at a single time point due to its retrospective nature. Fourth, Lp-PLA2 activity tests were not performed in this study. However, Lp-PLA2 activity is highly associated with vascular inflammation and the vulnerability of atherosclerotic plaque (41). At last, we could not exclude other effects on plaque vulnerability, such as dietary habits and environmental effects.

In conclusion, Lp-PLA2 may be a promising biomarker to predict plaque vulnerability in TIA patients with unilateral MCAs, especially in patients ≤ 60 years old. Testing of Lp-PLA2 as early as possible is necessary for patients with intracranial atherosclerotic stenosis.

## Data Availability Statement

The data that support the findings of this study are available from the corresponding author upon reasonable request.

## Ethics Statement

The studies involving human participants were reviewed and approved by Ethics Committees of The First Affiliated Hospital of Soochow University AND the Ethics Committees of The First People's Hospital of Kunshan. The patients/participants provided their written informed consent to participate in this study.

## Author Contributions

HZ: study design. YQ and SD: study concepts. YQ and XQ: data acquisition. XQ and XL: statistical analysis. JJ: data supervisor. YL, DW, and QZ: manuscript preparation. YQ, ML, and JX: language editing. YZ, SD, and HZ: manuscript review. All authors contributed to the article and approved the submitted version.

## Conflict of Interest

The authors declare that the research was conducted in the absence of any commercial or financial relationships that could be construed as a potential conflict of interest.
